# Mitigating End‐Stage Fatigue: Acute Inhaled Salmeterol Preserves Sprint Power in Simulated Cycling

**DOI:** 10.1111/sms.70159

**Published:** 2025-11-06

**Authors:** Michele Merlini, Walter Staiano, Luca Angius, Marco Romagnoli, Federico Schena, John Dickinson, Samuele Marcora

**Affiliations:** ^1^ School of Natural Sciences University of Kent Canterbury UK; ^2^ Faculty of Science of Physical Education and Sport University of Valencia Valencia Spain; ^3^ Department of Psychology University of Southern Denmark Odense Denmark; ^4^ Department of Sport, Exercise and Rehabilitation Northumbria University Newcastle Upon Tyne UK; ^5^ Department of Neuroscience, Biomedicine and Movement (DNBM) University of Verona Verona Italy; ^6^ Department of Life Quality Studies (QUVI) University of Bologna Bologna Italy

**Keywords:** β_2_‐agonists, doping, durability, elite sport, ergogenic aid, physiological resilience, WADA

## Abstract

This study tested whether a single 100 μg inhalation of salmeterol enhances 12‐s sprint performance in both fresh and fatigued states in elite road cyclists. In a randomized crossover design, 16 well‐trained, non‐asthmatic male cyclists completed 2 trials 1 week apart. Participants inhaled either 100 μg salmeterol or placebo 1h before testing. Each trial involved: an initial 12‐s sprint (fresh), a 1h race simulation (40%–95% peak power output) with heart rate, blood lactate concentration, and rating of perceived exertion (RPE) monitored, and a final 12‐s sprint (fatigued). Peak and mean power, and vastus lateralis myoelectric activity were recorded during the sprints. Power declined from pre‐ to post‐simulation in both conditions (*p* < 0.016), but the decrement was attenuated with salmeterol (peak: −7.5% vs. −18.2%; mean: −13.0% vs. −19.8%). Fatigued‐sprint peak power was higher with salmeterol (915 ± 135 W) than placebo (831 ± 112 W; *p* = 0.030), as was mean power (692 ± 76 vs. 643 ± 92 W; *p* = 0.037). No effect of salmeterol was observed on fresh sprint performance and myoelectric activity. Blood lactate concentration and RPE rose similarly in both conditions (*p* < 0.001), while heart rate was higher with salmeterol during the first 20 min (*p* = 0.004). Acute inhalation of salmeterol attenuates muscle fatigue and enhances sprint performance at the end of a simulated race. These findings challenge the presumption of no enhancing effect of inhaled salmeterol at therapeutic doses in competitive road cycling, where final sprints often determine outcomes.

## 1 Introduction

Approximately 22% of athletes experience lower airway dysfunction such as asthma or exercise‐induced bronchoconstriction (EIB) [1]. Athletes in sports that have high ventilatory demands and take place in challenging environments (cold, dry, polluted) have the highest prevalence. Most athletes with asthma and/or EIB will use a form of inhaled β_2_‐agonists as part of disease management to reverse or protect against airway smooth muscle bronchoconstriction. Athletes who compete in sports that sign up to the World Anti‐Doping Agency (WADA) code are only permitted to use four types of inhaled β_2_‐agonists: salbutamol (≤ 1600 μg in a 24 h period, ≤ 600 μg in an 8 h period), salmeterol (≤ 200 μg in a 24 h period), formoterol (≤ 54 μg in a 24 h period), and vilanterol (≤ 25 μg in a 24 h period). It is assumed that if athletes use permitted doses of inhaled β_2_‐agonists no ergogenic action will be elicited [2]. This assumption is corroborated by the results of the most recent meta‐analysis showing that β_2_‐agonists do not affect aerobic performance in non‐asthmatic subjects regardless of type, dose, administration route, duration of treatment or performance level of participants [3]. With regard to anaerobic performance, oral and supratherapeutic inhaled doses of β_2_‐agonists have been shown to improve muscle strength and sprint performance [4, 5, 6]. For instance, high‐dose inhaled terbutaline has been demonstrated to improve both muscle strength and repeated sprint performance in athletes [7]. However, inhaled formoterol at therapeutic doses has been reported to improve anaerobic performance, suggesting that even within approved dosing limits, some β_2_‐agonists may exert ergogenic effects [8]. For most β_2_‐agonists, when administered via inhalation at the low doses typically prescribed for asthma or EIB, systemic bioavailability remains limited, and the resultant impact on anaerobic performance is negligible [5].

Most studies investigating the acute effects of β_2_‐agonists on muscle strength and sprint performance have assessed these variables in a fresh, non‐fatigued state. A few exceptions include two studies [9, 10] that examined the acute effects of 800 or 1600 μg salbutamol inhalation on single and repeated sprint performance in football players, both in the fresh state and after fatigue induced by intermittent running protocols. These studies found no effect of this short‐acting β_2_‐agonist (SABA) on sprint performance under either condition. In contrast, Kalsen et al. [11] reported that administration of a 54‐μg dose of formoterol (a long‐acting β_2_‐agonist [LABA]) significantly increased knee extensor muscle strength measured before and after an all‐out 30‐s cycle ergometer test (Wingate Test). Moreover, the relative decline in muscle strength after the Wingate Test tended to be smaller with formoterol compared to placebo, suggesting mitigation of muscle fatigue. Analysis of the power output decline during the Wingate Test also indicated a positive effect of this LABA on fatigue resistance.

The importance of testing the effects of ergogenic aids not only in fresh states but also in fatigued ones is now becoming more appreciated in sports science, where the concept of fatigue resistance, now renamed as “physiological resilience” or “durability”, has been proposed as the fourth dimension of endurance performance [12, 13]. Of particular interest to us are road cycling races that involve multiple high‐power efforts, such as steep climbing, early breakaways, and final sprints, where managing fatigue accumulation across stages is critical to maintaining performance [14, 15, 16]. A study by Bedi et al. [17] found that an acute total dose of 360 μg of albuterol enhances severe‐intensity cycling performance by 23% compared to placebo (time to exhaustion 196 vs. 159 s) after 1 h of continuous heavy cycling exercise to mimic the physical demands of a road race in a group of competitive cyclists. To the best of our knowledge, there are no studies on the effects of β_2_‐agonists on sprint performance (power produced during sprints lasting between 9 and 17 s) [14] in a fatigued state in cyclists. Given the importance of sprint finishes in winning many cycling road races [14], further investigations are warranted.

To fill this gap, we investigated whether a single 100 μg dose of inhaled salmeterol enhances sprint performance in both fresh and fatigued conditions in elite male road cyclists. Fatigue was induced by cycling for 1h at increasing submaximal power outputs to simulate end‐stage demands. The secondary aims of this study were to investigate the acute effects of this LABA on myoelectric activity of the vastus lateralis during the sprints and on the cardiometabolic and perceptual responses during the race simulation.

## 2 Materials and Methods

### 2.1 Participants

A convenience sample of 16 elite male road cyclists [mean ± SD: age 25 ± 1.7 years; height 180 ± 6 cm; body mass 75.7 ± 8.4 kg; V̇O_2_max 64.6 ± 6.9 mL·kg^−1^·min^−1^; peak power output (PPO) during the incremental V̇O_2_max test 419 ± 38 W; > 5 sessions·week.^−1^; > 400 km·week.^−1^; ≥ 5 y competitive experience] was recruited and classified as Level 4 athletes [18]. All were healthy, free of medications and demonstrated no EIB, confirmed by a negative eucapnic voluntary hyperventilation (EVH) challenge [9]. A sensitivity power analysis using GPower3.1.9.7 (Heinrich‐Heine‐Universität, Düsseldorf) revealed that a sample size of 16 for a 2 × 2 repeated measure ANOVA (*α* = 0.05; power = 0.80; number of groups = 1; number of measurements = 4; correlation among repeated measures = 0.8; nonsphericity correction *ε* = 1) is sufficient to detect an effect size f = 0.19 that corresponds to *η* ^*2*^ *p* = 0.03 (small to moderate effect). The protocol was approved by the University of Valencia ethics committees (Approval No. 1057/1071521) and conformed to the declaration of Helsinki. Participants provided informed consent, received standardized procedural instructions, but remained naïve to study hypotheses.

### 2.2 Equity, Diversity, Inclusion

The main eligibility criteria were being a competitive cyclist between 18 and 45 years of age, with no current diseases or disabilities. No participants were excluded according to ethnicity, race, color, religion, sex, sexual orientation, gender identity, national origin, or other individual status.

### 2.3 Design and Procedures

A double‐blind, crossover, counterbalanced, randomized experimental design was used. Participants visited the laboratory on four separate occasions. On visit 1, participants were screened for bronchoconstriction. In short, after baseline spirometric assessment and a 5 km time trial, forced expiratory volume in 1‐s (FEV_1_) was measured in duplicate at 3, 5, 10, 15, 20, and 30 min post exercise, recording the higher value; a ≥ 10% fall in FEV_1_ post exercise was defined EIB [19]. During Visit 2, participants performed an incremental test (50 W increments every 2 min to voluntary exhaustion) on an Excalibur Sport cycle ergometer (Lode, NL) to determine V̇O_2_max (MetaLyzer 3B, Cortex Biophysik, DE) and PPO. Five minutes later, with the cycle ergometer in isokinetic mode at 110 RPM [20], participants were familiarized with the 12‐s all‐out sprint test. Power data were corrected for flywheel acceleration and averaged over 1‐s intervals.

Visits 3 and 4 implemented the experimental trials. Before each trial, athletes rated motivation for the upcoming task on a 5‐point Likert scale [21], then inhaled either 100 μg salmeterol (2 × 50 μg Serevent Accuhaler, GSK, UK) (SAL) or a matched placebo (PLA). One hour post inhalation, they performed a standardized warm‐up (5 min at 100 W, free cadence), followed by three 12‐s submaximal “ramp” sprints at 25%, 50%, and 75% of max effort, each separated by 0.5–5 min active recovery [22]. Next, a true 12‐s all‐out sprint (“Pre”) was executed; capillary blood was sampled immediately thereafter and analyzed for lactate concentration (Lactate Pro LT‐1710, Arkray, JP). Athletes then commenced a 1‐h race simulation scaled to the individual PPO measured during the V̇O₂max test: 20 min at 40%, 15 min at 45%, 15 min at 55%, 5 min at 65%, 4 min at 75%, and a final 1 min at 95% PPO. This protocol was designed to simulate the physical demands of sprinting at the end of a stage during professional road cycling races [14]. Lactate was measured again at minutes 35 and 59. Heart rate (HR) (Polar S610i, Polar, FI), rating of perceived exertion (RPE) using the Borg 6–20 scale [23], and the cadence freely chosen between 80 and 100 RPM, were recorded at minutes 1, 5, 10, 15…59, 60.

Immediately following the race simulation, the cycle ergometer was returned to isokinetic mode, and participants produced a second 12 s all‐out sprint (“Post”), replicating the sprint finish of a road race [12]. Lactate was measured for the fourth and final time immediately after Post. Verbal encouragement was provided during all sprints by the same researcher blind to treatment order allocation. Peak and mean power, and fatigue index [FI = (peak power − minimum power)/peak power] were computed for each sprint. During the sprints, surface EMG (10 mm Ag/AgCl electrodes, 20‐mm inter‐electrode spacing) recorded vastus lateralis activity [24]. EMG signals were amplified and sampled at 2 kHz with 16‐bit resolution. During post‐processing analysis, EMG signals were filtered with a Butterworth band‐pass filter (10–500 Hz) and processed via root mean square (RMS) with a 100‐ms interval window [22, 25]. Three consecutive crank revolutions (the second, third and the fourth) during isokinetic mode were identified and averaged. The peak and mean RMS were used as indices of myoelectric activity during the 12‐s sprint at Pre and Post in both conditions.

### 2.4 Statistical Analysis

All data are reported as mean ± SD unless noted. Normality and sphericity assumptions were assessed via Shapiro–Wilk and Mauchly's tests, with Greenhouse–Geisser correction applied as needed. Fully repeated‐measures 2 × 2 ANOVAs (time: pre vs. post; condition: SAL vs. PLA) evaluated sprint performance (peak and mean power, and FI) and EMG metrics (peak and mean RMS). Separate 2 × 14 ANOVAs assessed the effect of condition on HR, RPE and cadence during the race simulation, while 2 × 4 ANOVAs examined blood lactate concentration across sampling points. Paired *t*‐tests were used to compare motivation scores between conditions. Significant interactions prompted Bonferroni‐adjusted post hoc tests. In the absence of significant interactions, only the main effects of time and condition were reported. Alpha was set at 0.05 (two‐tailed) for all analyses. Effect sizes for ANOVAs are partial *η* ^*2*^ (small = 0.02, moderate = 0.13, large = 0.26). Ninety five percentage confidence intervals of the differences between SAL and PLA were calculated for the sprint peak and mean power at pre and post. Analyses were performed in SPSS 27.

## 3 Results

There was no significant difference in motivation scores between SAL (3.41 ± 0.32 AU) and PLA (3.27 ± 0.25 AU) (*p* = 0.42).

**FIGURE 1:**
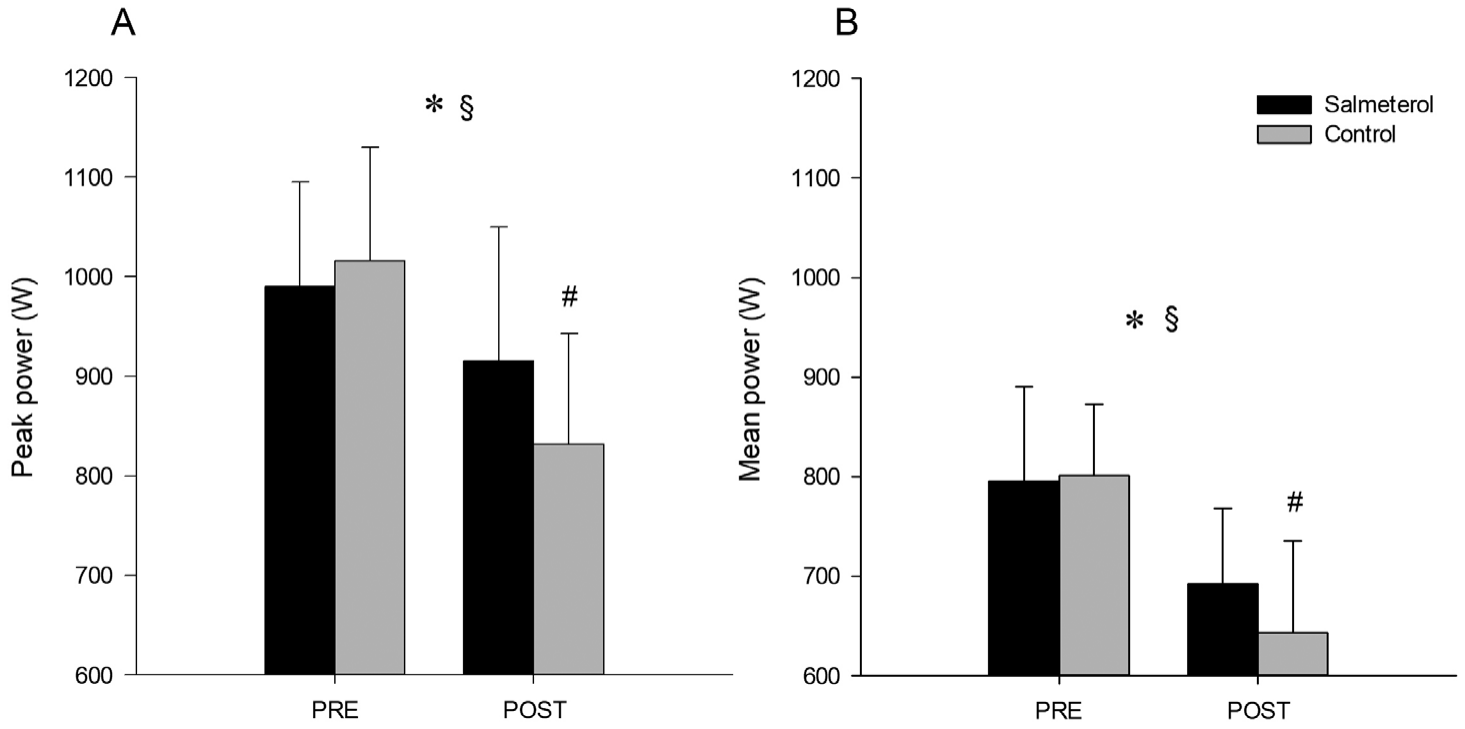
Effect of treatment on PPO (A) and mean power output (B) before (pre) and after (post) the 1‐h race simulation. (*) significant main effect of time. ($) significant condition × time interaction. (#) simple main effect of condition according to Holm–Bonferroni method. Data are presented as means ± SD.

With regards to sprint performance, there was a significant time × condition interaction for peak power (*p* = 0.016, *η* ^*2*^ *p* = 0.424) (Figure 1A). Post hoc tests revealed no significant difference in peak power at pre between SAL (995 ± 105 W) and PLA (1016 ± 114 W) (*p* = 0.088); 95% CI = −21.1 (−51.1 to 9.12). In both SAL (*p* = 0.016) and PLA (*p* < 0.001), there was a significant decline in peak power between pre and post. However, such a decline was smaller in SAL so that peak power at post (915 ± 135 W) was significantly higher than in PLA (831 ± 112 W) (*p* = 0.030); 95% CI = 84.3 (7.40 to 163). There was also a significant time × condition interaction for mean power (*p* = 0.01, *η* ^*2*^ *p* = 0.470) (Figure 1B). Post hoc tests revealed no significant difference in mean power at pre between SAL (795 ± 95) and PLA (801 ± 72) (*p* = 0.646); 95% CI = −6.33 (−35.8 to 23.1). In both SAL (*p* < 0.001) and PLA (*p* < 0.001) there was a significant decline in mean power between pre and post. However, such a decline was smaller in SAL so that mean power at post (692 ± 76 W) was significantly higher than PLA (643 ± 92 W) (*p* = 0.037); 95% CI = 48.3 (−0.771 to 95.9). No condition × time interaction (*p* = 0.948, *η* ^*2*^ *p* = 0.000) and no main effect of condition (*p* = 0.556, *η* ^*2*^ *p* = 0.021) were found for the FI. The FI increased significantly between pre (31.5 ± 6) and post (37.2 ± 8) (main effect of time *p* = 0.023, *η* ^*2*^ *p* = 0.300).

Analysis of surface EMG of the vastus lateralis found no time × condition interactions for peak RMS (*p* = 0.566, *η* ^*2*^ *p* = 0.021) and mean RMS (*p* = 0.601, *η* ^*2*^ *p* = 0.028). While neither myoelectric activity indices differed by condition (peak RMS *p* = 0.090, *η* ^*2*^ *p* = 0.019; mean RMS *p* = 0.364, *η* ^*2*^ *p* = 0.021), both decreased significantly between Pre and Post (main effect of time peak RMS *p* = 0.015, *η* ^*2*^ *p* = 0.319; main effect of time mean RMS *p* = 0.035, *η* ^*2*^ *p* = 0.372).

**FIGURE 2:**
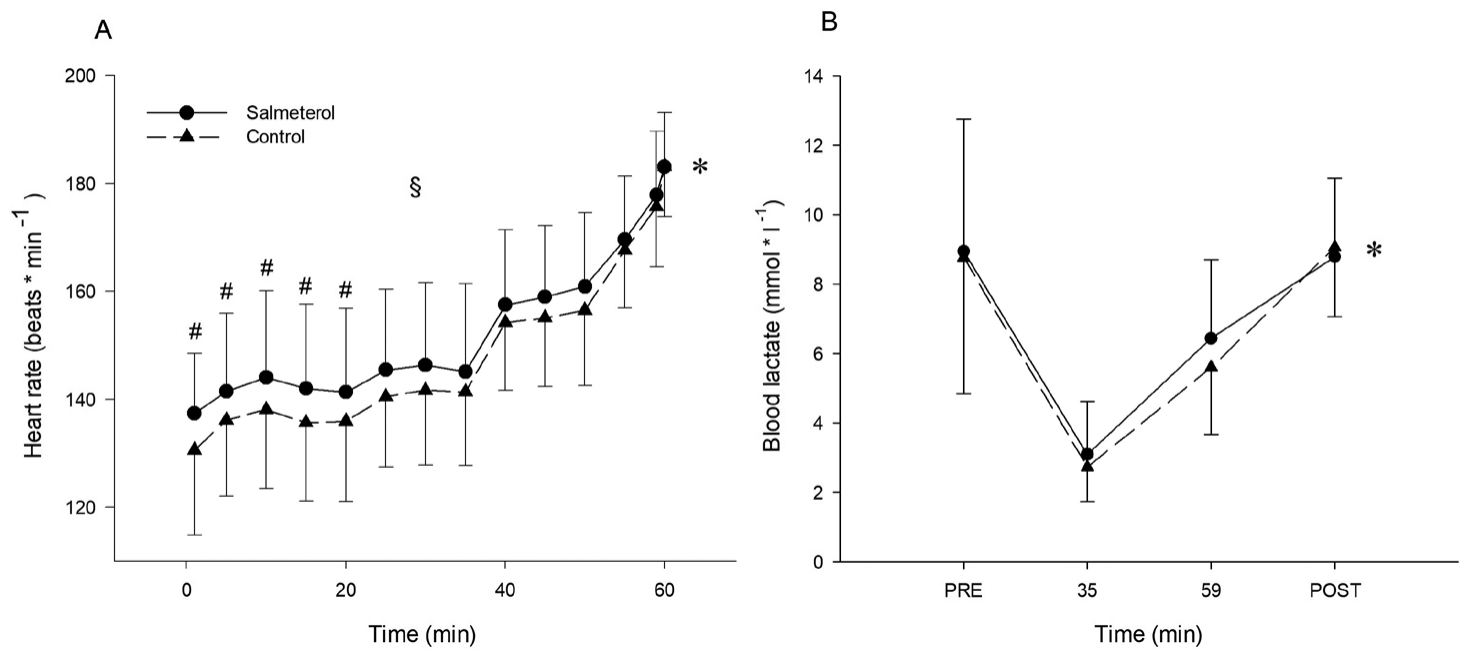
Effect of condition on physiological responses during the 1‐h race simulation. Heart rate (A) and blood lactate concentration (B). (*) significant main effect of time. ($) significant condition × time interaction. (#) simple main effect of condition according to Holm‐Bonferroni method. Data are presented as means ± SD.

**FIGURE 3:**
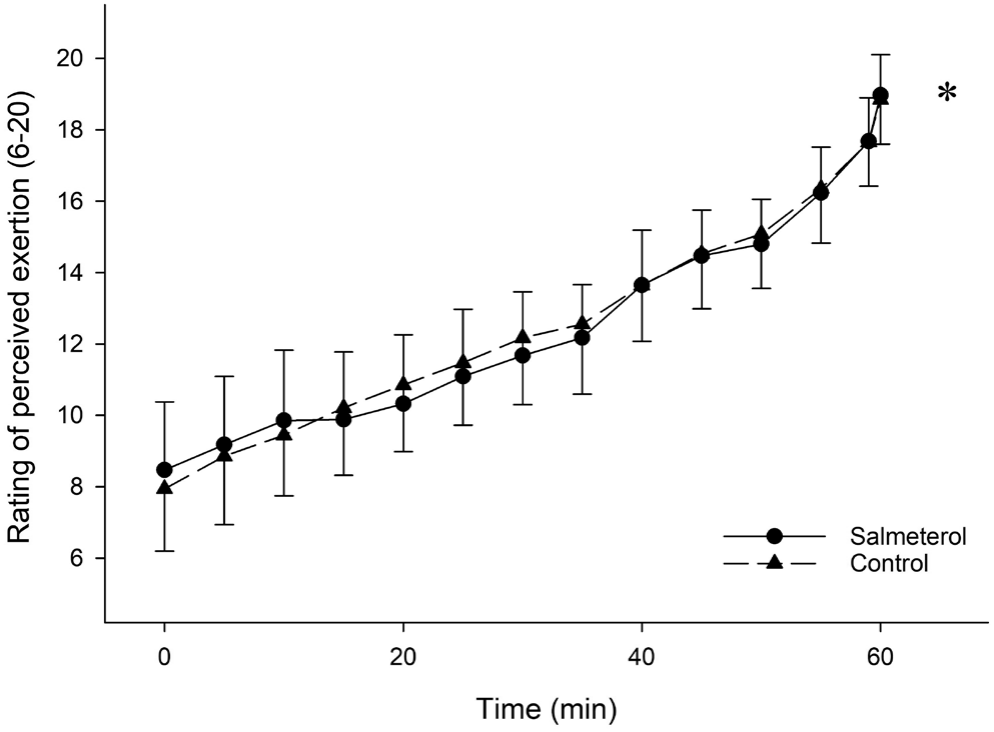
Effect of condition on rating of perceived exertion (RPE) during the 1‐h race simulation. (*) significant main effect of time. Data are presented as means ± SD.

Pedal cadence during the race simulation remained stable (grand mean: 89 ± 4) across both condition and time with no interaction (all ps > 0.05, *η* ^*2*^ *p* < 0.030). With regard to the responses during the race simulation, HR exhibited a significant time × condition interaction (*p* = 0.004, *η* ^*2*^ *p* = 0.211) (Figure 2A). Post hoc tests revealed that HR rose progressively and significantly in both conditions (all ps < 0.037 against 1 min). However, HR was higher in SAL compared to PLA at minutes 1 (*p* = 0.041), 5 (*p* = 0.049), 10 (*p* = 0.043), 15 (*p* = 0.038), and 20 (*p* = 0.026). RPE increased over time similarly in both conditions (main effect of time *p* < 0.001, *η* ^*2*^ *p* = 0.933) with no main effect of condition (*p* = 0.802, *η* ^*2*^ *p* = 0.004) nor interaction (*p* = 0.323, *η* ^*2*^ *p* = 0.069) (Figure 3). Blood lactate concentration rose markedly across sprints and the race simulation (main effect of time *p* < 0.001, *η* ^*2*^ *p* = 0.724) but did not differ by condition (*p* = 0.540, *η* ^*2*^ *p* = 0.028) and no interaction was present (*p* = 0.480, *η* ^*2*^ *p* = 0.049) (Figure 2B).

## 4 Discussion

In the fresh state, a single inhalation of 100 μg salmeterol did not produce a statistically significant effect on sprint performance in elite male road cyclists. However, under fatigued conditions, inhaled salmeterol appeared to reduce fatigue‐related declines in sprint performance.

These findings in the fresh state align with some previous studies [4, 5], that found no clear evidence of an ergogenic effect on sprint performance when using WADA‐approved doses of β_2_‐agonists. With specific reference to salmeterol, McDowell et al. [26] also observed no improvement in 30‐s sprint performance in elite non‐asthmatic track cyclists three hours after inhalation of 42 μg salmeterol. Similarly, Morton et al. [27] found no effect of 50 μg of salmeterol on power output during 10 and 30‐s cycling sprints in non‐asthmatic cyclists and triathletes. However, our findings are in contrast with Jeppesen et al. [8] which showed an ergogenic effect of WADA‐permitted doses of formoterol on sprinting performance in fresh states in male cyclists.

Importantly, in our study, salmeterol significantly reduced the fatigue‐related decline in sprint performance observed after a 1‐h cycling protocol designed to mimic the demands of a sprint finish in professional road racing. Compared with placebo, salmeterol resulted in a smaller reduction in peak power (7.5% vs. 18.2%) and mean power (13.0% vs. 19.8%) during the final 12‐s sprint (post) relative to baseline (pre), indicating that salmeterol mitigates end‐stage fatigue. Because of this effect of salmeterol, peak and mean power during the final 12‐s sprint were 84 W and 49 W higher, respectively, than in the placebo condition. These differences are highly relevant to real racing conditions because they correspond to the 6%–10% differences in peak and mean power considered sufficient to influence the outcome of a sprint finish [28]. Indeed, during sprint finishes, peak power in the first ~2–3 s determines acceleration and initial positioning, whereas mean power sustained over 10–20 s determines the ability to maintain velocity and resist being overtaken before the finish line [28].

These ecologically valid findings in cyclists align with those of a laboratory study by Kalsen et al. [11], who reported that the administration of a 54‐μg dose of formoterol significantly mitigated the muscle fatigue (measured as the decline in maximal knee extensor strength) induced by a Wingate Test in a group of recreationally active male subjects. However, in another study from the same laboratory, high‐dose terbutaline exacerbated the muscle fatigue induced by two 45‐s bouts of cycling exercise at 560 ± 11 W with 4 min of recovery in between bouts [29]. In this study, maximal knee extensor strength in fatigued conditions did not differ between high‐dose terbutaline and placebo [29]. In two earlier studies [9, 10] we investigated the acute effects of 800 or 1600 μg salbutamol inhalation on single and repeated sprint performance in football players fatigued by intermittent running protocols. In these studies, we found no beneficial effect of salbutamol on sprint performance in a fatigued state. Variations in the type, dose, and administration route of the β_2_‐agonists used in these studies, as well as differences in testing and fatiguing protocols, and participant characteristics, may explain these discrepant findings.

With regard to the potential mechanisms underlying the reduction in muscle fatigue observed in the present study, salmeterol did not significantly affect task‐related motivation and myoelectric activity of the vastus lateralis. These results suggest that salmeterol did not reduce central fatigue. Therefore, the most likely explanation for the ergogenic effects of salmeterol on peak and mean power produced during the final 12‐s sprint (fatigued condition) is peripheral. These speculations are corroborated by previous reports of unchanged neural and neuromuscular responses following acute β_2_‐agonist administration [30, 31, 32]. On the contrary, at the peripheral level, there is evidence that acute β_2_‐agonist administration may mitigate muscle fatigue by increasing the rates of glycogenolysis and glycolysis during sprinting in humans [7]. Furthermore, β_2_‐agonists can improve K^+^ handling, enhance Ca^2+^ release and speed up Ca^2+^ re‐uptake [33]. Together, these peripheral effects may explain why β_2_‐agonists can temporarily enhance sprint power in conditions of depleted muscle glycogen [34] and altered ionic homeostasis induced by fatiguing cycling exercise [33].

In the present study, a single 100 μg inhaled dose of salmeterol had limited effects on the cardiometabolic and perceptual responses during the 1 h cycling protocol simulating the final part of a road race. Heart rate was modestly elevated under salmeterol in the initial stages (1–20 min) but converged with placebo responses at higher workloads (≥ 40% PPO). Similar effects have been found in previous studies. For example, Fleck et al. [35] found that the acute inhalation of 360 μg albuterol increases HR at 200, 225, and 250 W of incremental cycling exercise to exhaustion (∼400 W) in a group of competitive male road cyclists. More recently, Koch et al. [36] observed, in a similar group of trained male cyclists, a higher HR during a 10 km time trial performed after inhalation of 1600 μg salbutamol, despite similar power output during the time trial compared to placebo. These findings most likely reflect the well‐known chronotropic effect of β_2_‐agonists [37]. However, this effect is not universal. In elite cyclists, Helge et al. [38] found no acute effect of 800 μg salbutamol inhalation on HR measured during a long cycling protocol consisting of 150 min of cycling at 60% of PPO interspersed, at min 30 and 110, by a 15 min increment (5 min at 65%, then 5 min at 70% and again 5 min at 65% of PPO) before returning to cycling at 60% of PPO.

Blood lactate concentration immediately after the two sprints (in both fresh and fatigued states) and during the race simulation did not differ significantly between conditions in the present study. Our results are in agreement with previous findings during submaximal cycling exercise [35, 38] and after short, all‐out cycling exercise when lactate is measured immediately after the sprint [11, 39]. In these studies, no consistent effect of β_2_‐agonists on lactate responses was found. However, β_2_‐agonists seem to increase blood lactate concentration when samples are taken several minutes after the sprint [5, 8, 11]. This effect of β2‐agonists may reflect higher rates of glycogenolysis and glycolysis during sprinting [7].

Despite the mitigation of locomotor muscle fatigue, we failed to find a significant effect of salmeterol on perceived fatigue defined as the progressive increase in perceived effort that occurs during endurance exercise [40]. Indeed, RPE during the race simulation did not differ from placebo. This finding is consistent with the study of Fleck et al. [35] who found no effect of acute inhalation of 360 μg albuterol on RPE during an incremental cycling test to exhaustion in a group of competitive male road cyclists. No acute effect of β_2_‐agonists on perceived fatigue in elite cyclists was also found by Helge and colleagues [38] who measured RPE during a long cycling protocol after inhalation of 800 μg salbutamol. Given the importance of perceived effort in limiting and predicting endurance performance [40], our findings are consistent with most of the literature that shows no significant effects of β_2_‐agonists on endurance performance [3, 5].

This study has several limitations that temper the interpretation of its findings. First, the sample size was small (16 male cyclists), which limits statistical power for detecting modest performance effects and reduces generalizability to broader athlete populations. Second, no female athletes were included, so sex‐specific pharmacokinetic or pharmacodynamic differences remain unaddressed. Third, dosing was not adjusted for body mass; all participants received a fixed 100 μg salmeterol dose, which could introduce variability in systemic absorption and effect (a lighter athlete may experience a higher relative dose and drug concentration than a heavier athlete). Finally, this acute trial did not examine long‐term salmeterol use, so the potential for β_2_‐agonists adrenoceptor desensitization or downregulation with chronic administration, and any resultant attenuation of drug efficacy, remain unknown.

## 5 Perspective

While β₂‐agonists at WADA‐approved dosages appear to fail to boost endurance performance in non‐asthmatic athletes [3, 5], our study provides a significant advance: A single 100 μg dose of inhaled salmeterol reduces locomotor muscle fatigue and improves peak and mean power during a 12‐s sprint performed by elite cyclists after a one‐hour cycling protocol simulating the demands of a sprint finish in professional road racing. The observed effects are practically relevant because they correspond to the 6%–10% differences in peak and mean power considered sufficient to influence the outcome of a sprint finish [18]. These findings suggest that permitted salmeterol doses under current WADA guidelines may mitigate end‐stage fatigue and provide a significant advantage during a sprint finish. These results carry important implications: they challenge assumptions underlying therapeutic use exemptions and doping thresholds, and they prompt further research into the acute and chronic effects of salmeterol and more generally of LABAs and SABAs on performance in fatigued conditions. If replicated, our findings may influence future revisions of anti‐doping policies regarding inhaled salmeterol.

## Author Contributions

**Michele Merlini:** methodology, software, validation, formal analysis, writing – original draft, visualization, supervision. **Walter Staiano:** software, validation, formal analysis, resources, data curation, Writing – original draft, writing – review editing, visualization, supervision. **Luca Angius:** software, validation, investigation, data curation, writing – review editing, visualization. **Marco Romagnoli:** software, data curation, writing – review editing, supervision, project administration. **Federico Schena:** methodology, software, formal analysis, writing – review editing, visualization, supervision, project administration. **John Dickinson:** methodology, validation, investigation, resources, writing – review editing, visualization. **Samuele Marcora:** conseptualization, methodology, validation, resources, writing – review editing, supervision, project administration.

## Conflicts of Interest

The authors declare no conflicts of interest.

## Data Availability

The data that support the findings of this study are available on request from the corresponding author. The data are not publicly available due to privacy or ethical restrictions.
